# Helping Healthcare to Help Itself: A Response to the Recent Commentaries

**DOI:** 10.34172/ijhpm.2023.8384

**Published:** 2024-01-07

**Authors:** Paul Holmström, Thomas Björk-Eriksson, Fredrik Bååthe, Caroline Olsson

**Affiliations:** ^1^Department of Medical Radiation Sciences, Institute of Clinical Sciences, Sahlgrenska Academy at Gothenburg University, Gothenburg, Sweden; ^2^Regional Cancer Centre West, Gothenburg, Sweden; ^3^Department of Oncology, Institute of Clinical Sciences, Sahlgrenska Academy, Gothenburg University, Gothenburg, Sweden; ^4^LEFO – Institute for Studies of the Medical Profession, Oslo, Norway; ^5^Institute of Stress Medicine, Gothenburg, Sweden; ^6^Sahlgrenska University Hospital, Gothenburg, Sweden; ^7^Institute of Health and Care Sciences, Sahlgrenska Academy at Gothenburg University, Gothenburg, Sweden

 We thank *International Journal of Health Policy and Management* for inviting so many learned researchers to comment on *our paper*^1^ and in turn thank them for their thoughtful and valuable comments. As several of them are referenced in *the paper*, it is particularly interesting to take part of their views.

 As noted by Brailsford,^2^ *the paper *bridges the main author’s previous experience as a management consultant and his research journey in system dynamics (SD), through a masters, a licentiate and finally a doctoral degree. Given the lack of implementation discussed earlier by Brailsford et al,^3^ the aim of the resulting research became to scientifically explore Holmstrom’s many change and improvement cases in healthcare in a systematic way to inductively create learnings from the rich empirical material.

 In retrospect it is embarrassing that, as observed by Brailsford,^2^ “patient centred care” is part of many cases of *the paper*, yet actual patients were not involved. We can only acknowledge the relevance of Argyris^4^ work as to the gap between what people say (espoused theory) and what people do (theory-in-use).

## Language

*The paper* is dense as academic papers are. Øvretveit^5^ kindly provides additional context helping readers unfamiliar with the subjects. We agree with Øvretveit that there is a risk that SD approaches may be too complicated for most practitioners, in particular group model building, where participants are directly involved in model building. In the cases described in *the paper*, participants mainly interacted with a graphical user interface showing inputs and outputs of the model. The problem clarification phase in Figure 4 builds entirely on the terminology of the participants. In several cases, patient flow diagrams were used to map the descriptions of the group before “translating” it into a SD model, which was built iteratively, gaining relevance and acceptance by each step.

## Pragmatically Mixing Methods

 Ackerman^6^ asks if clear designs were produced in advance. In each case, a project proposal outlined meeting plans and work flows. As they typically were adhered to, the plans were not repeated in the case descriptions. The sequencing in the proposals differed depending on problem descriptions and learnings from previous projects. In addition, actual flows were pragmatically adapted to the facilitator’s perception of “where the group was.” This corresponds well to Noto^7^ stating that both SD and action research (AR) fit well within the pragmatism research philosophy in addressing real-world issues.

 Øvretveit^5^ also notes the usefulness of the SD/AR combination leading to more sophisticated understanding of systems for practitioners. Zolfagharian^8^ seems to agree, emphasizing striking similarities between SD and AR approaches that make their integration promising as they complement each other. He writes that SD contributes by eliciting, capturing, and changing mental models. AR by transforming the social reality and changing the mindset of stakeholders. We completely agree with this.

## Problem Structuring Methods

 Ackermann^6^ found it challenging that there is little recognition, in *the paper*, of the work done in the field of problem structuring methods. We realize that *the paper* has not managed to clearly convey the importance of problem identification and structuring in the five described cases. As described in *the paper* and the enclosed case descriptions, all cases began with exhaustive listing of problems and objectives. Interconnectedness between issues were explored, leading to agreed naming and causal loop diagrams. The entire first divergent and convergent phase in Figure 4 in *the paper* concerns problem clarification and structuring.

## Pluralistic Groups and Complexity

 Zolfagharian^7^ observes that group composition in the cases seems to be pluralistic, in respect to Coalesced Authority, Power, and Influence. He writes that the mixed research design can converge the divergences in the opinions of the participants about the problems as well as their resolutions. Zolfagharian notes that although members of a pluralistic group do not share the same values and beliefs, their basic interests are compatible. However, Wehrens et al^9^ state that it is unlikely that stakeholders can converge on a mutually developed point of reference, as they have conflicting values.

 We agree that differences between professions need to be considered. This has often been done using stakeholder theory. Both SD and AR are considered as suitable for that purpose. Gratton^10^ describes such major differences as fault lines and found that they are best addressed by focusing on the task at hand rather than people. Menzies^11^ classical text on hospital care discusses the emotional demands and defences of individuals working in healthcare settings, by first focusing on task, then on people.

*The paper* describes pragmatic task-oriented processes, which use causal loop diagrams and SD models as neutral devices. A model does not take the part of any stakeholder, it will not reflect reality until all important perspectives have been included. *The paper* describes it as if participants bring their jigsaw pieces to the process as shapeholders rather than stakeholders. By focusing on creating a useful model, differences between professions are complementary rather than confrontational. In our experience, this can lead to a model becoming a mutually developed point of reference.

 Any change of procedure requires working together. One may have to abandon established routines for new routines that are perceived as uncertain. The modelling process reduces uncertainty. In his commentary Øvretveit^5^ notes that change projects can be time consuming and take practitioners away from other work. Our experience is that pragmatic time-efficient projects stimulate engagement across professions, when focused on improving their own work processes.

## Implementation

 Brailsford^2^ notes that there is remarkably little evidence of successful implementation of model results. She comments that while *the paper* reports the short-term outcomes of each project, the longer-term outcomes are only reported for case study 2. In his PhD thesis,^12^ Holmström writes that specific projects, where external consultants or researchers are engaged, usually are embedded in larger contexts. When a project is concluded, learnings are carried forward in future stages and new projects. The early AR researchers Emery and Trist^13^ wrote “A main problem in the study of organizational change is that the environmental contexts in which organizations exist are themselves changing.” Therefore, it may be difficult to assess the usefulness of any model results as they become blended with what happened before the project was initiated and after it was completed. In all the described cases, other interventions were made afterwards, such as re-organization, organizational development, or further research. Even as to case 2, the obstetrics and maternity ward was closed two years later, and staff transferred to another hospital.

 In the journey of learning and organizational change, completeness is elusive. We, therefore, suggest moving from measuring implementation rates to assessing learnings. The true measure of success lies not only in what is achieved but in the power of continuous learning, making a model a potent transitional object in the dynamic voyage of growth and knowledge. This position is reflected in the quote by the Swedish Nobel laureate Tranströmer: “You’ll never be complete, and that’s as it should be.”

## Acknowledgements

 We particularly wish to thank all authors of commentaries to our original article

## Ethical issues

 Not applicable.

## Competing interests

 Authors declare that they have no competing interests.
